# Displacement and force distribution of splinted and tilted mandibular anterior teeth under occlusal loads: an in silico 3D finite element analysis

**DOI:** 10.1186/s40510-016-0129-x

**Published:** 2016-06-01

**Authors:** Allahyar Gerami, Sepideh Dadgar, Vahid Rakhshan, Puya Jannati, Farhad Sobouti

**Affiliations:** Department of Orthodontics, Dental Faculty, Tehran University of Medical University, Tehran, Iran; Department of Orthodontics, Dental Faculty, Mazandaran University of Medical Sciences, PO Box: 19551-624, Sari, Iran; Department of Dental Anatomy and Morphology, Dental School, Azad University, Tehran, Iran; Iranian Tissue Engineering and Research Center, Tehran University, Tehran, Iran; Student Research Committee, Faculty of Dentistry, Mazandaran University of Medical Sciences, Sari, Iran

**Keywords:** Lower anterior teeth, Tooth inclination, Retention, Orthodontic treatment, Splinting, Finite element method

## Abstract

**Background:**

Fixed orthodontic retainers have numerous advantages, but it is not known whether they can exert pathological forces on supporting tissues around the splinted teeth. The purpose of this study was to investigate how the inclination of the lower anterior teeth can affect dental displacement and also change the direction of occlusal loads exerted to dental and its supporting tissues.

**Methods:**

Four three-dimensional finite element models of the anterior part of the mandible were designed. All the models contained the incisors and canines, their periodontal ligament layers (PDLs), the supporting bone (both spongy and cortical), and a pentaflex splinting wire placed in the lingual side of the teeth. Teeth inclination was considered to be 80° (model 1), 90° (model 2), 100° (model 3), and 110° (model 4) to the horizontal plane. The lower incisors were loaded with a 187-N vertical force. Their displacement patterns and the stress in their PDLs were evaluated.

**Results:**

In incisors with 80° of inclination, less than a 0.1-mm lingual displacement was seen on the incisal edge and a similar distance of displacement towards the labial was seen on their root apices. However, in models with 90°–110° of inclination, the incisal edge displaced labially between about 0.01 and 0.45 mm, while root apices displaced lingually instead. By increasing the angle of the teeth, the strain in the periodontal ligament increased from about 37 to 58 mJ. The von Mises stresses around the cervical and apical areas differed for each tooth and each model, without a similar pattern. Increasing the angle of the teeth resulted in much higher cervical stresses in the incisors, but not in the canines. In the lateral incisor, cervical stress increased until 100° of inclination but reduced to about half by increasing the angle to 110°. Apical stress increased rather consistently in the incisor and lateral incisors, by increasing the inclination. However, in the canines, apical stress reduced to about half, from the first to fourth models.

**Conclusions:**

Increasing the labial inclination can mostly harm the central incisors, followed by the lateral incisors. This finding warns against long durations of splinting in patients with higher and/or patients with reduced labial bone thickness.

## Background

The stability of orthodontic treatment outcome is a major clinical concern, since many cases especially mandibular anterior teeth relapse after aligning [1, 2]. Permanent or long-term retention seems to be the only way to provide a proper post-treatment alignment [2, 3]. A proper method for this purpose is to use fixed retainers that remain permanently in the mouth and are invisible, compliance-free, and well tolerated [2, 4]. Fixed retainers were commonly made of stainless steel round wires and later thinner coaxial or braided round wires; among various fixed retainers of different metals, diameters, and designs, the flexible spiral wire (twisted steel wire) is very popular between orthodontists for providing acceptable long-term retention [1, 2, 4–8].

Advantages of fixed retainers in relapse control are well documented in the literature. Despite their popularity, their adverse effects remain unclear. Their negative effects are a matter of controversy for many years, and they are regarded as a rather unpleasant strategy from a periodontist’s perspective because of plaque accumulation and hygiene control problems [8–15]. The question remaining to be answered is whether fixed retainers have biomechanical disadvantages, because their biomechanical aspects have never been evaluated numerically [8]. The extent of tooth displacement and also the distribution of occlusal force exerted on periodontal tissues of the retained teeth are not known. This is crucial especially when assuming that the post-treatment inclination of the mandibular teeth varies depending on the protocol of treatment. Patients with extraction treatment plans might have more upright teeth, while those with non-extraction treatments might have mandibular teeth tilted labially. Since masticatory forces are exerted in a vertical direction, inclination of teeth might make the masticatory forces more hazardous for teeth inclined labially than for those positioned more upright and parallel to the force direction. Therefore, the purpose of this study was to quantify the degree of mandibular teeth’s movement and changes in stress distribution around supporting tissues of mandibular anterior teeth splinted by a pentaflex wire with four different labiolingual inclinations (incisal mandibular plane angle (IMPA) = 80°, 90°, 100°, and 110°).

## Methods

**Fig. 1:**
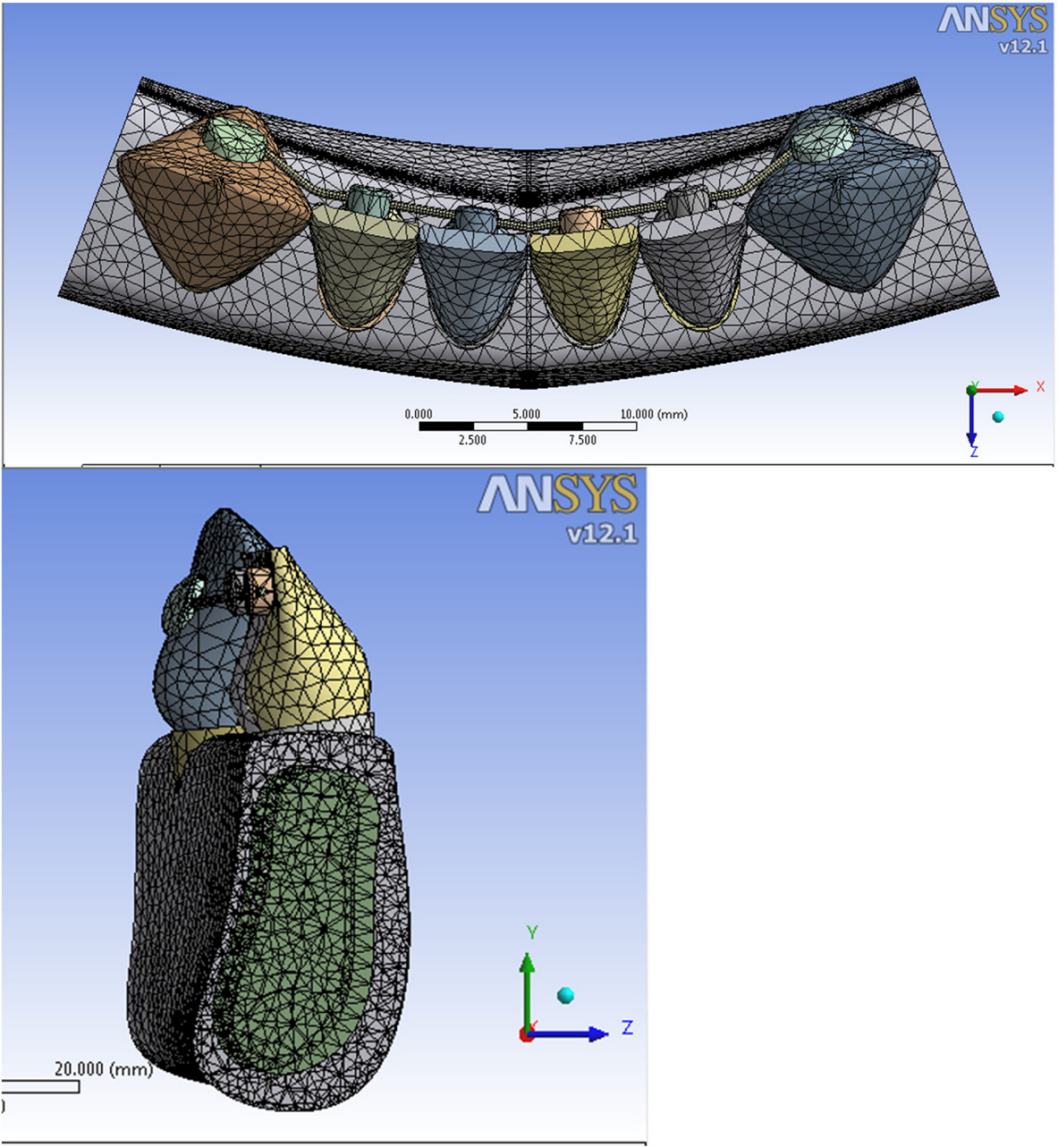
Meshed model 1 showing the inclination of the anterior teeth at 80° to the horizontal plane

**Fig. 2:**
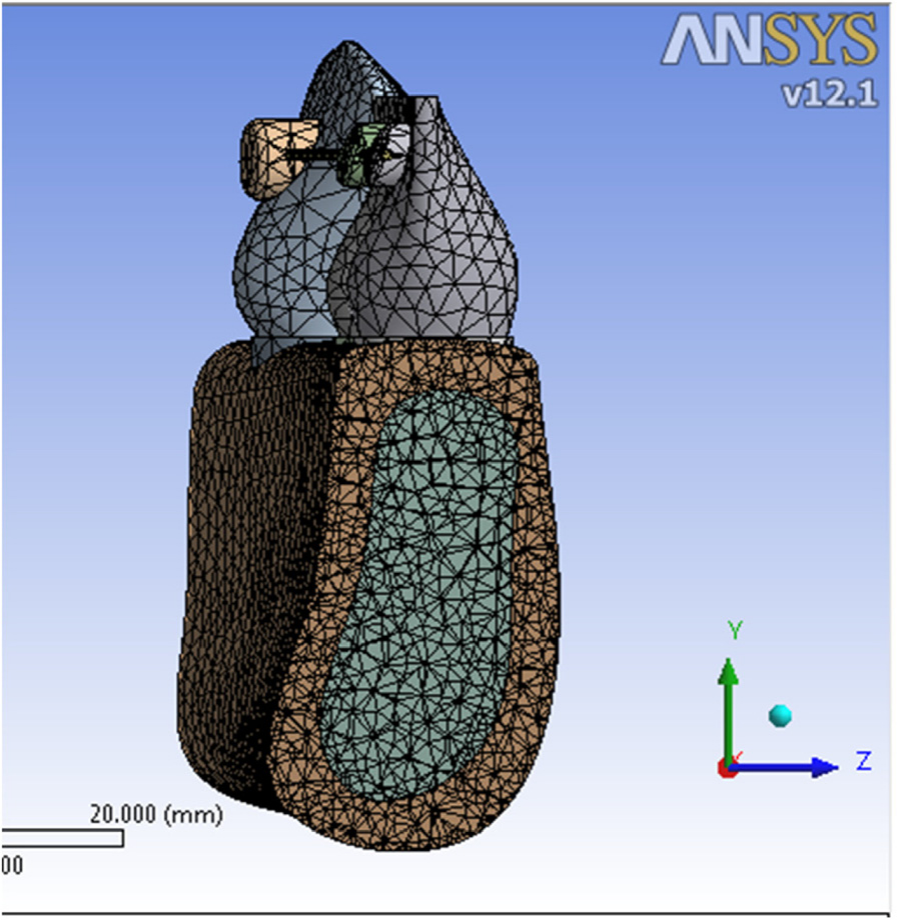
Meshed model 2 showing the inclination of the anterior teeth at 90° to the horizontal plane

**Fig. 3:**
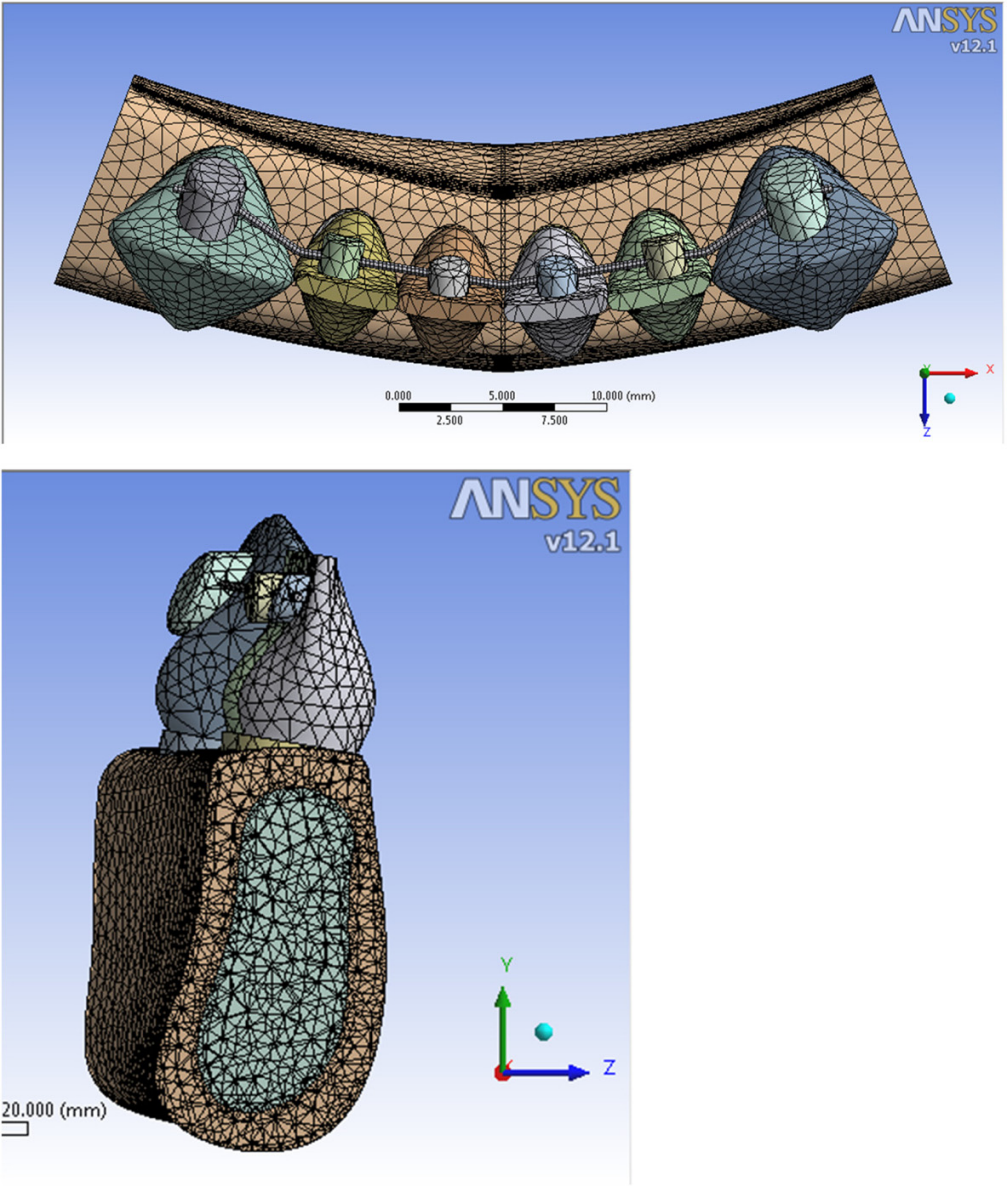
Meshed model 3 showing the inclination of the anterior teeth at 100° to the horizontal plane

**Fig. 4:**
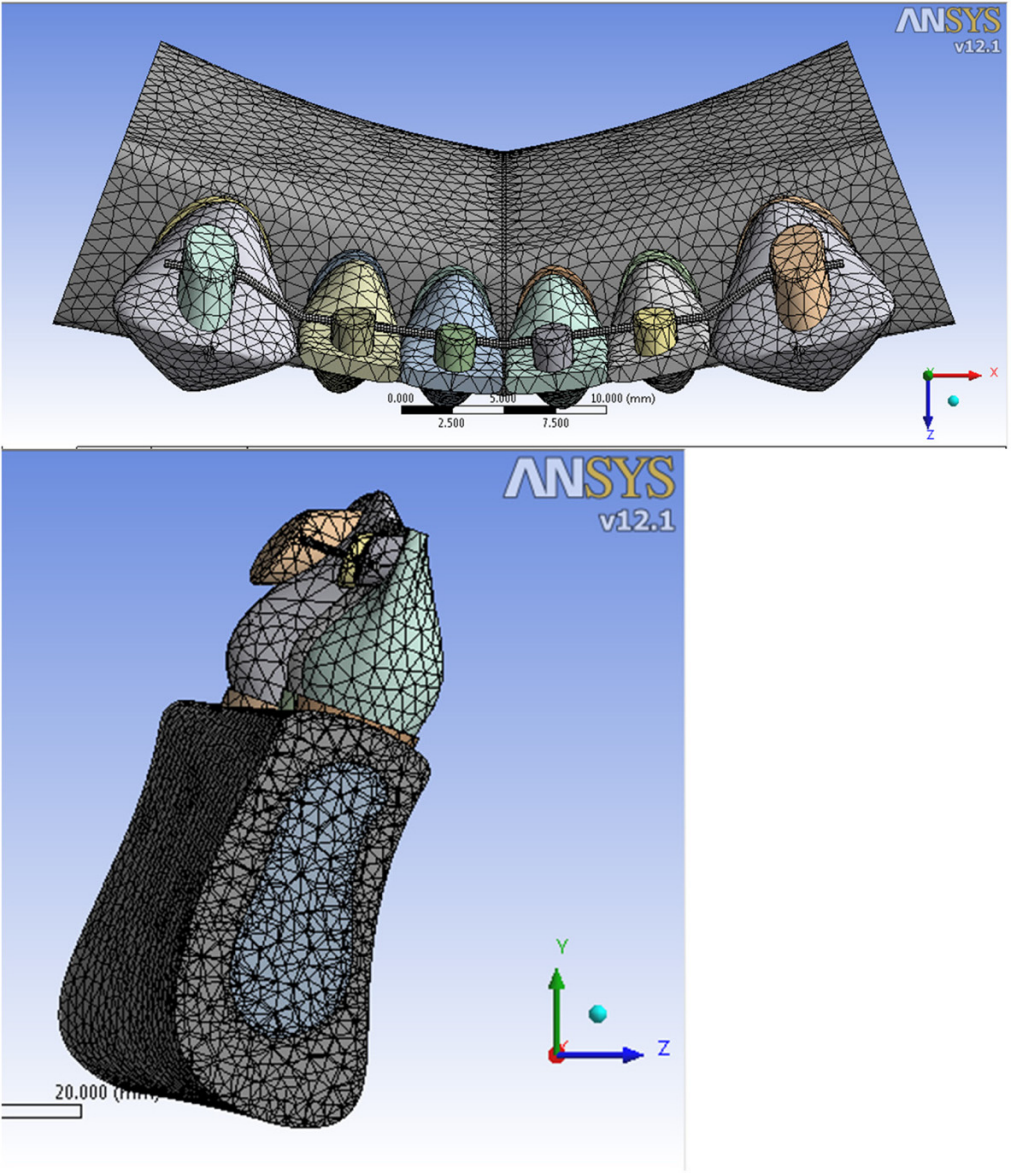
Meshed model 4 showing the inclination of the anterior teeth at 110° to the horizontal plane

Four 3D finite element models were designed of a mandibular anterior segment. It included six anterior teeth with the average dimensions and supporting structures [8, 16]. Each model consisted of a cancellous bone surrounded by a 1-mm-thick cortical layer. A simplified 0.25-mm-thick periodontal ligament layer (PDL) was modeled based on the root-form geometry of the teeth [8, 17]. All models had a bonded fixed retainer in the lingual surface of the anterior teeth. The models were similar except for the angle of the lower incisors to the horizontal plane. The inclination of the lower incisors to the horizontal plane was 80° in model 1, 90° in model 2, 100° in model 3, and 110° in model 4 (Figs. 1, 2, 3, and 4, respectively).

SolidWorks 2014 (300 Baker Ave. Concord, MA 01742, USA) was selected for the modeling phase. The models were then transferred to the ANSYS Workbench Ver. 11.0 (ANSYS Inc., Southpointe, 275 Technology drive, Canonsburg PA 15317, USA) for calculation [8, 17]. All the vital tissues were presumed elastic, homogeneous, and isotropic. The corresponding elastic properties such as Young’s modulus and Poisson’s ratio were applied (Table 1). The relationship between the teeth, their PDL, the spongy and cortical bones, and the multi-strand wire with composite and the teeth was provided by contact elements (Figs. 1, 2, 3, and 4). All rigid body motions were prevented. A vertical force of 187 N (as an average occlusal force usually exerted on the lower incisors) was applied at each incisal edge of the central incisors [8, 18, 19]. Tooth displacements in labial and gingival direction, the energy increase in the PDLs of the anterior teeth, and the von Mises stress in the cervical and apical parts of the PDLs were assessed.

**Table 1:** Mechanical properties of the materials used in modeling

	Young’s modulus (MPa)	Poisson’s ratio
Tooth [8]	20,300	0.26
PDL [8]	0.667	0.49
Composite [8]	16,600	0.24
Spongy bone [8, 17]	13,400	0.38
Cortical bone [8, 17]	34,000	0.26
Pentaflex wire [8]	90,000	0.3

## Results

### Tooth displacement

**Fig. 5:**
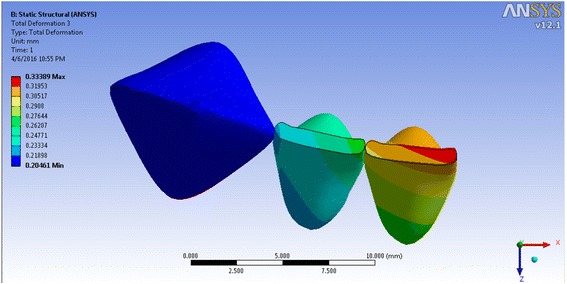
Total deformation in the teeth of the first model (the inclination of the anterior teeth at 80° to the horizontal plane)

**Fig. 6:**
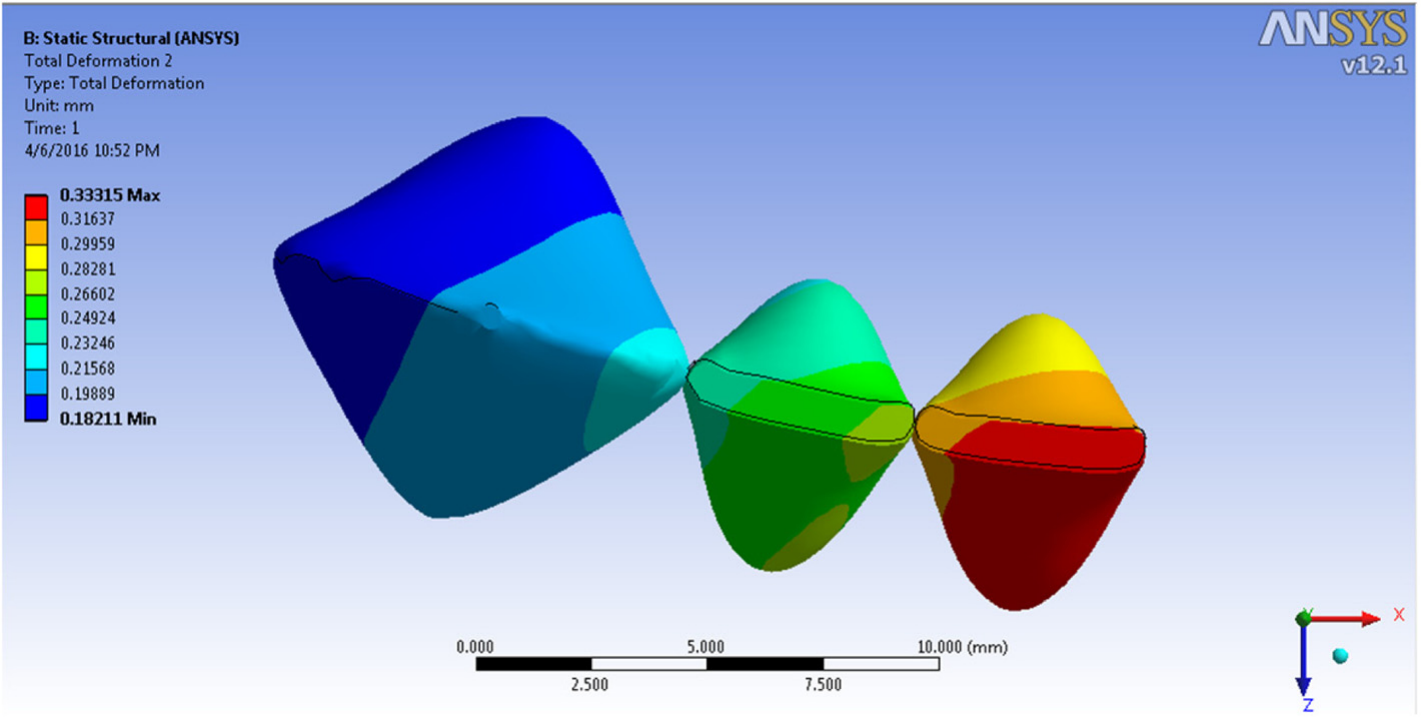
Total deformation in the teeth of the second model (the inclination of the anterior teeth at 90° to the horizontal plane)

**Fig. 7:**
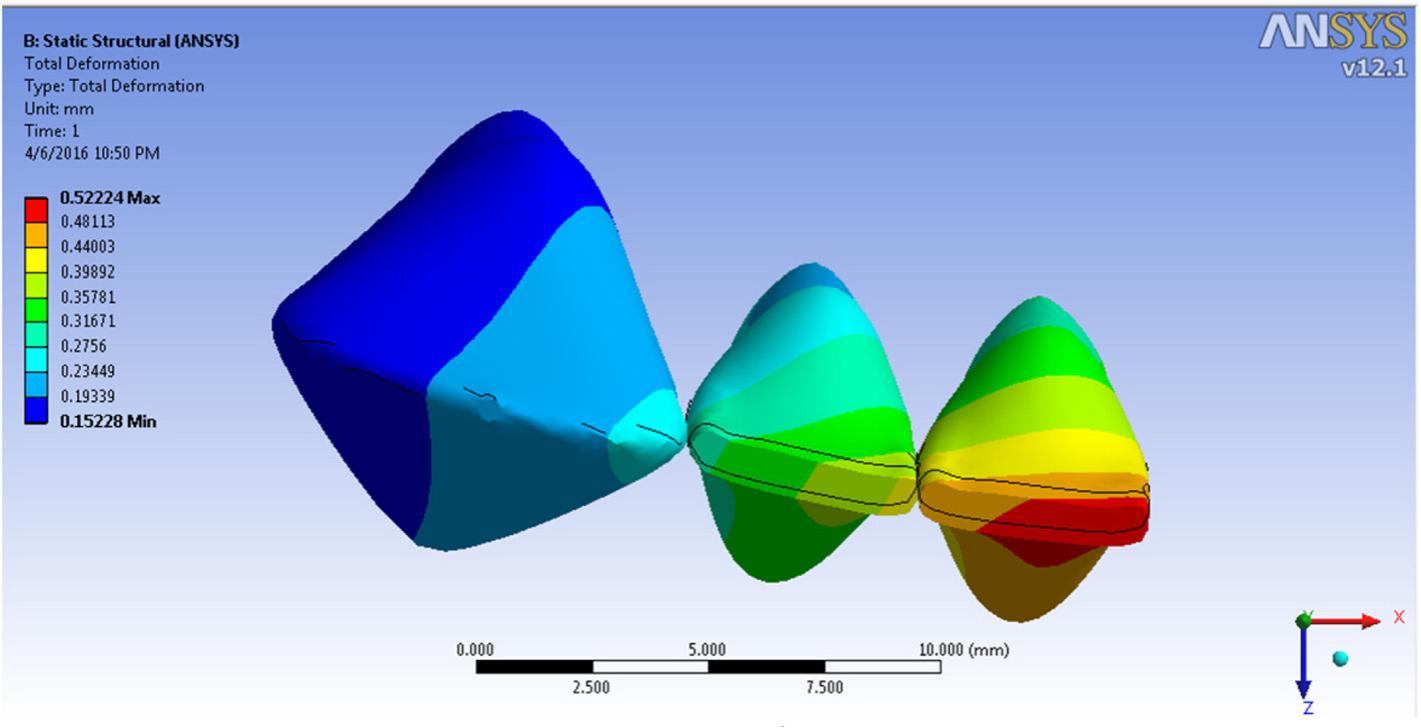
Total deformation in the teeth of the third model (the inclination of the anterior teeth at 100° to the horizontal plane)

**Fig. 8:**
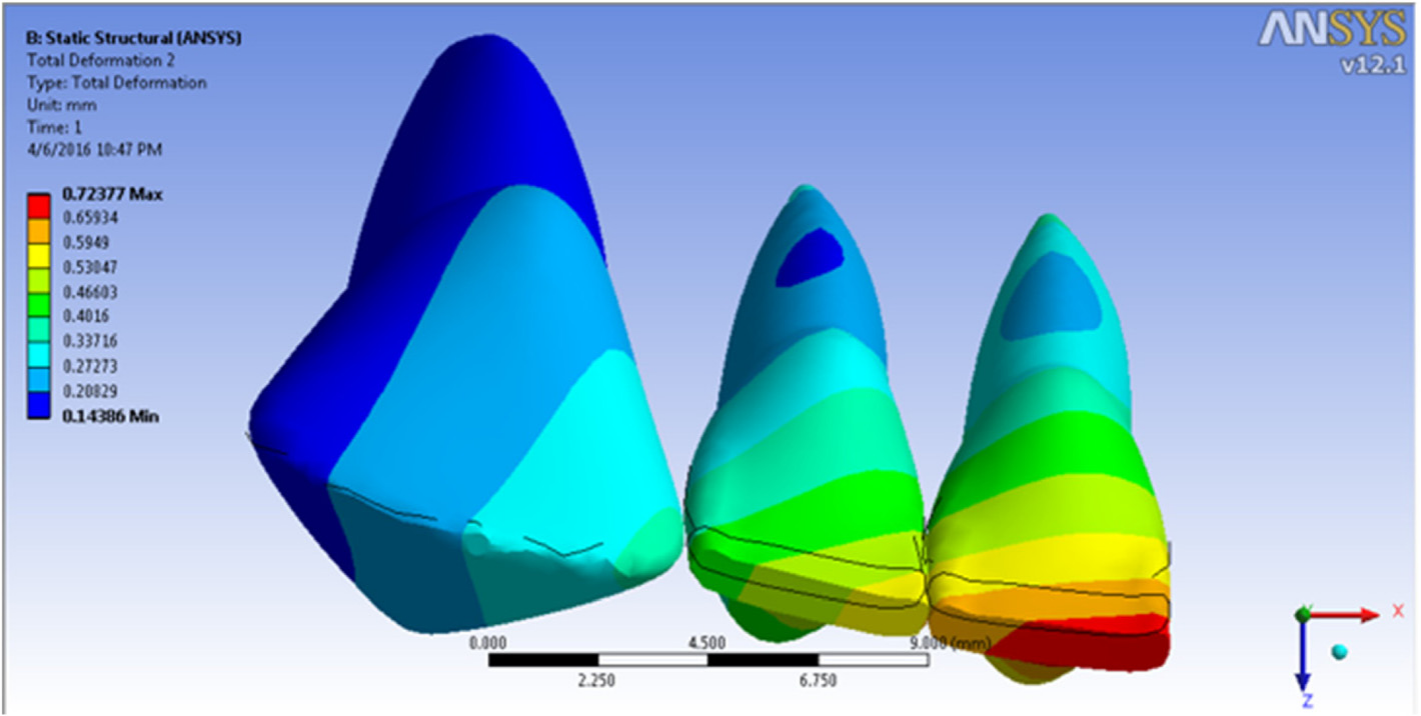
Total deformation in the teeth of the fourth model (the inclination of the anterior teeth at 110° to the horizontal plane)

The incisor displacement was −0.0725 mm (towards lingual) in the incisal edge and 0.0800 mm (towards labial) in the apical area, in model 1. The incisal edge movement turned to labial in models 2–4 (between 0.00939 and 0.4538 mm in the incisal edge) and between −0.00477 and −0.3119 mm in the apical area (Figs. 5, 6, 7, and 8; Table 2). Almost the same pattern is followed by the lateral incisor. The pattern of lingual displacement of the cusp tip (−0.0725 mm) and labial displacement in the apex (=0.008 mm) in model 1 was observed in the canine (Table 2).

**Table 2:** The incisal and apical displacements of the anterior teeth in various models

		Model 1 (80°)	Model 2 (90°)	Model 3 (100°)	Model 4 (110°)
Central incisor	Incisal	−0.07250	0.09390	0.28871	0.45381
	Apical	0.08000	−0.04770	−0.19580	−0.31195
Lateral incisor	Incisal	−0.02040	0.08130	0.20380	0.33305
	Apical	0.05000	−0.03560	−0.13740	−0.23122
Canine	Incisal	−0.07250	0.02740	0.08260	0.07580
	Apical	0.08000	−0.01090	−0.06520	−0.01430

### The von Mises stress in cervical and apical areas

**Fig. 9:**
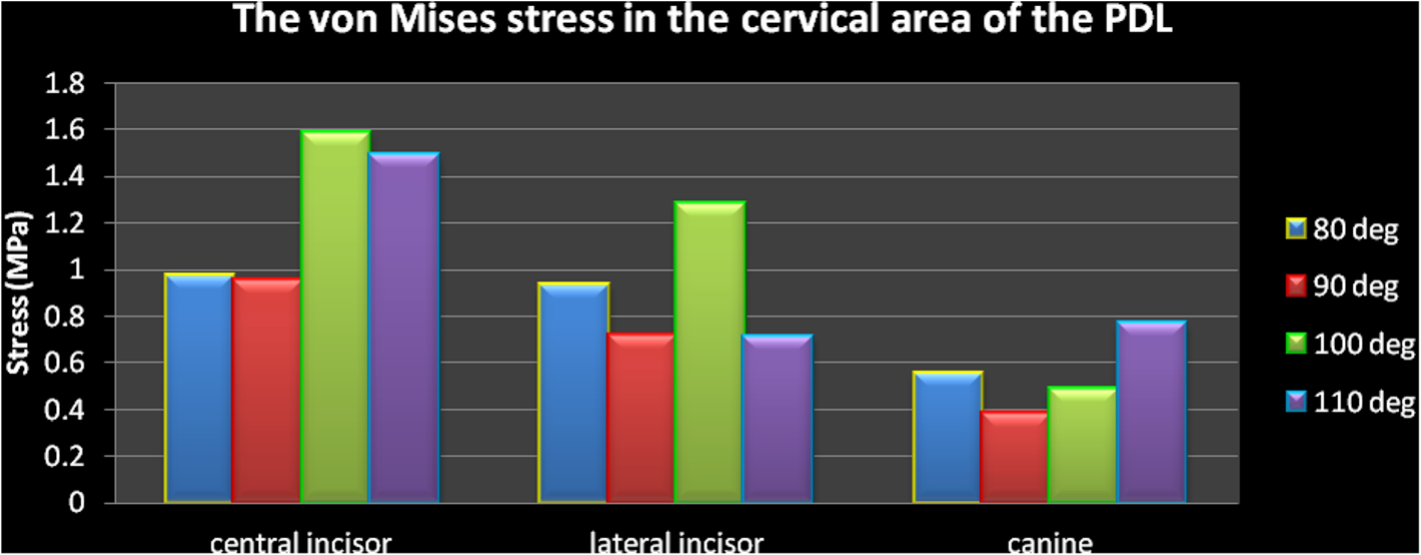
The von Mises stress in the cervical area of the PDL

**Fig. 10:**
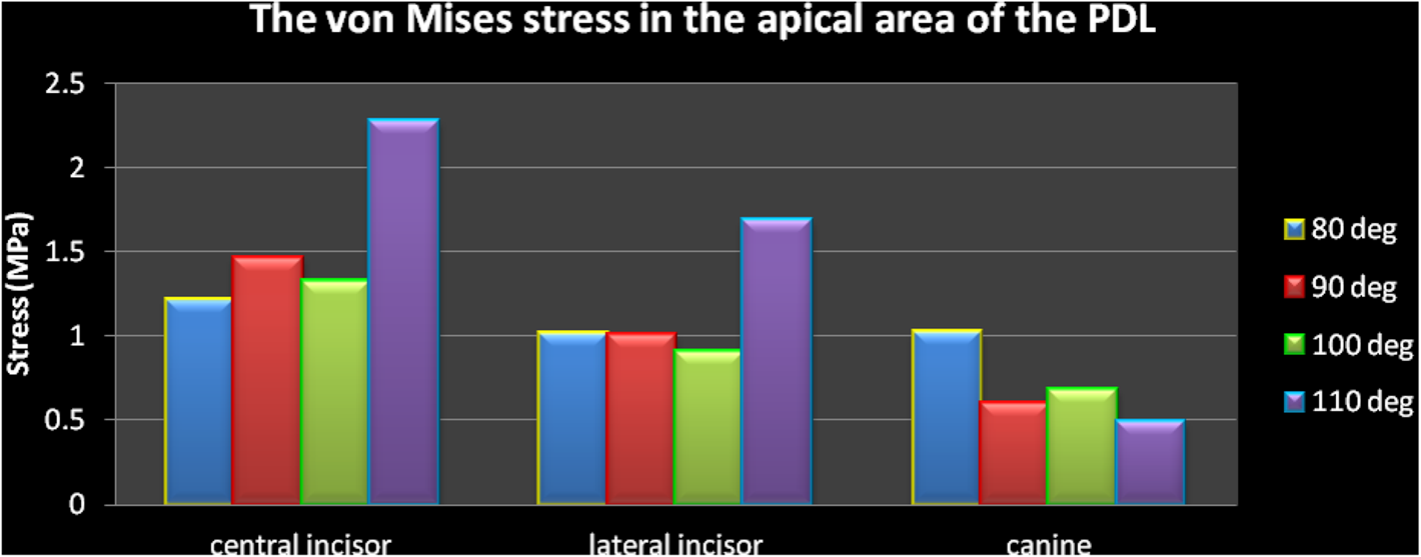
The von Mises stress in the apical area of the PDL

In all models, the stresses are higher in the apical area compared to the cervical part. The numeric findings are presented in Table 3 and Figs. 9 and 10. The canine findings are noticeable.

**Table 3:** The von Mises stress (MPa) in the PDL of the anterior teeth

		Model 1 (80°)	Model 2 (90°)	Model 3 (100°)	Model 4 (110°)
Cervical	Central incisor	0.97731	0.95656	1.5886	1.4942
	Lateral incisor	0.93226	0.72064	1.2836	0.71427
	Canine	0.5551	0.38212	0.48572	0.76753
Apical	Central incisor	1.2173	1.4611	1.3266	2.2735
	Lateral incisor	1.0151	1.00949	0.90826	1.6879
	Canine	1.0218	0.59451	0.67781	0.48768

### Strain energy

**Fig. 11:**
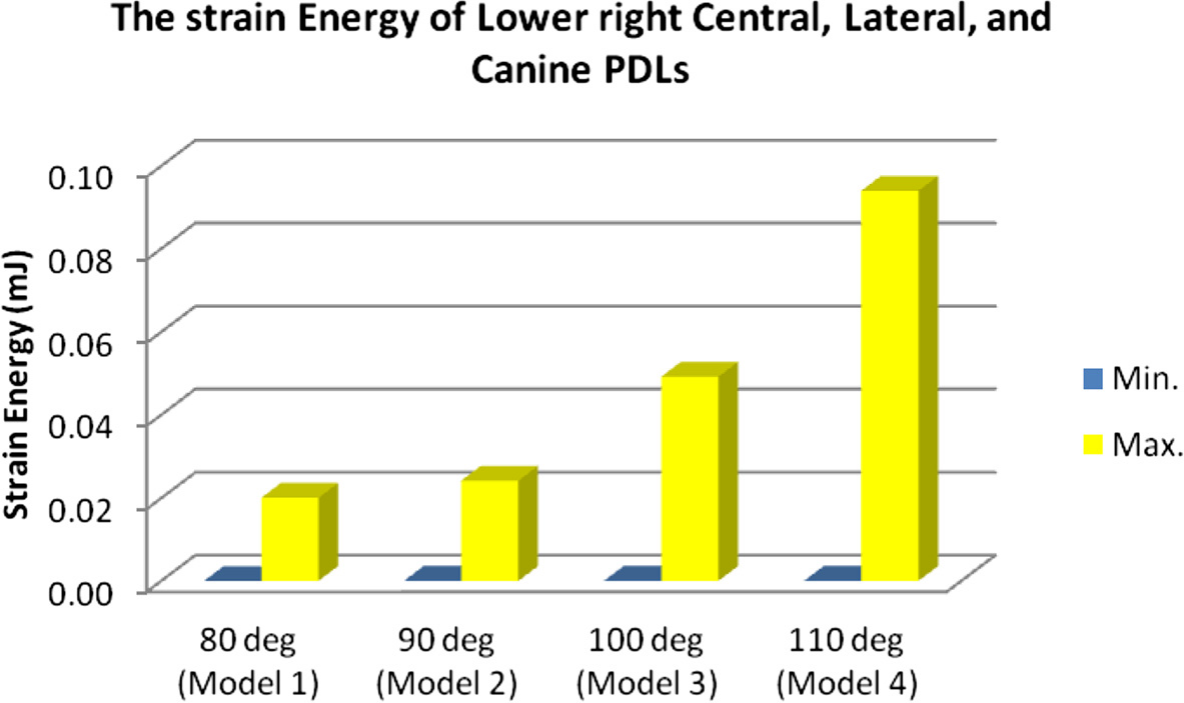
The strain energy of the lower right central, lateral, and canine PDLs

**Fig. 12:**
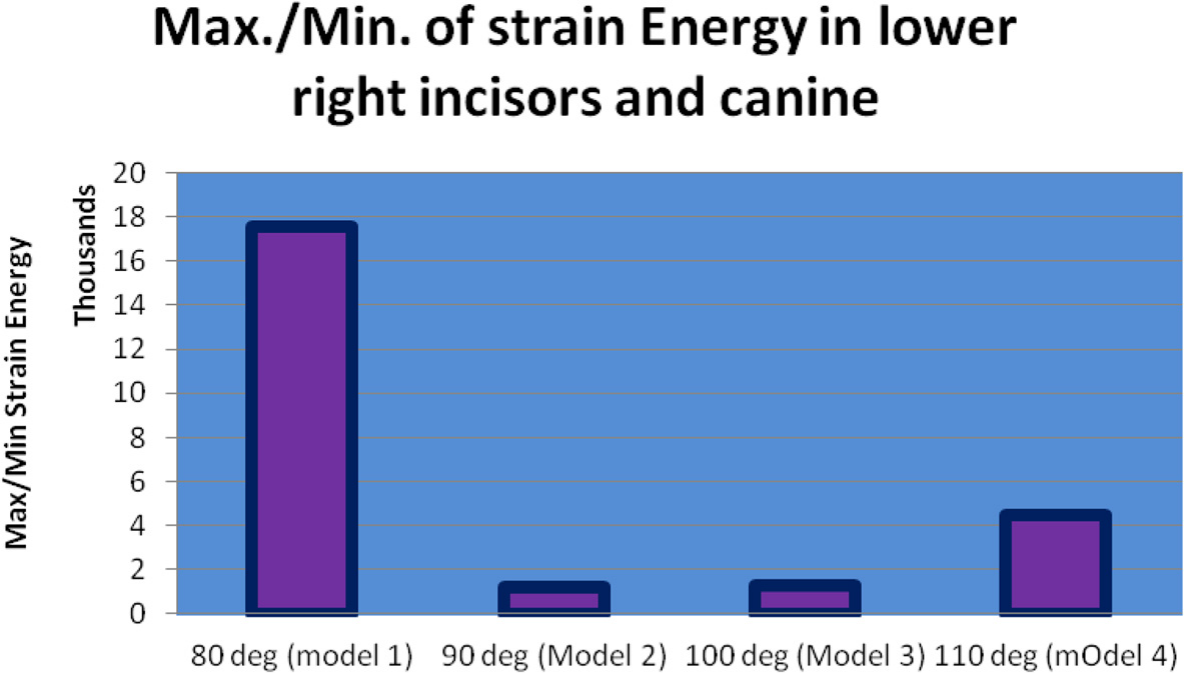
Max./Min. of strain energy in the lower right incisors and the canine

**Fig. 13:**
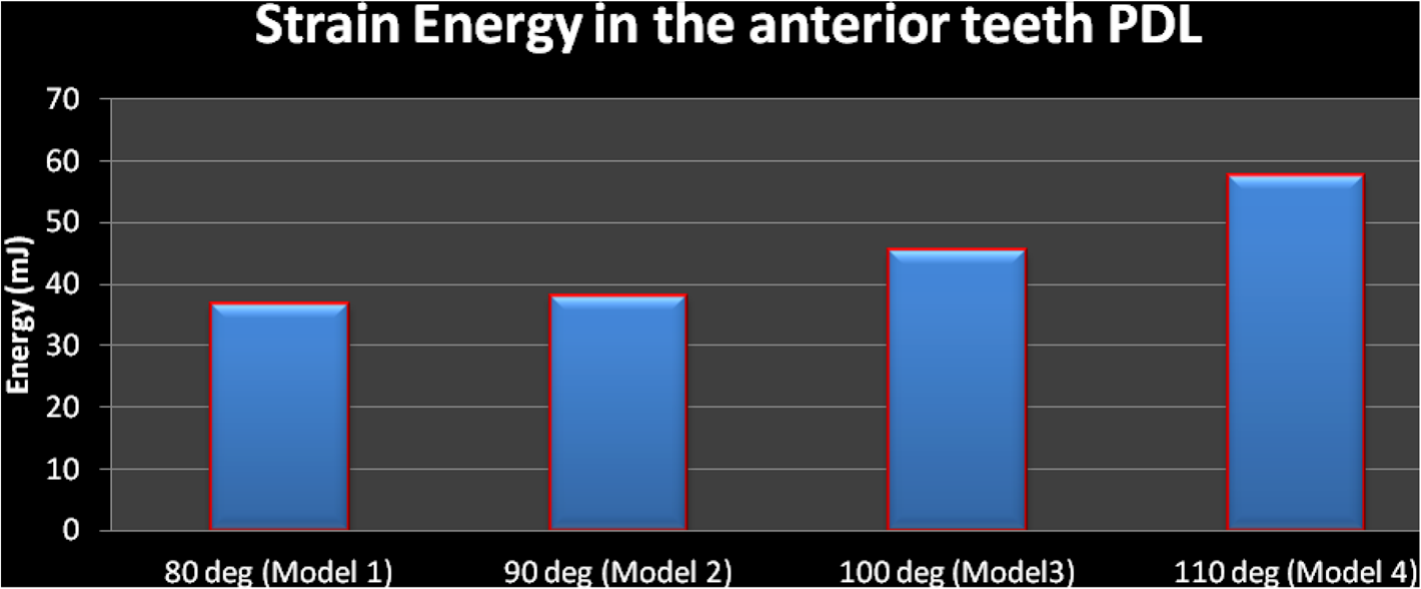
Strain energy in the anterior teeth PDL

The strain energy of the anterior teeth PDL was 36.734 mJ in model 1 and increased to 37.874 mJ in model 2. This increase was noticed in model 3 (45.28) and model 4 (57.502, Figs. 11, 12, and 13).

## Discussion

The viscoelastic nature of periodontal tissues plus adaptations in the anatomic characteristics like the bone mass and level and the width of the periodontal ligament space are the key to the physiologic tooth mobility [13, 20]. The wire in a fixed retainer can undergo elastic deflection by being mechanically deformed under masticatory loads [13, 20]. In an average male patient, the bite force can increase up to 113 N, which might cause mechanical deformation of the retainer [21]. It is desirable for the teeth not to be fixed in too rigid positions during the orthodontic retention period [4, 5].

In this study, in the incisors with 80° of inclination, less than a 0.1-mm lingual displacement was seen on the incisal edge and a similar distance of displacement towards the labial was seen on the root apices. However, in models 2 to 4 (with 90° to 110° of inclination), the incisal edge displaced labially between about 0.01 and 0.45 mm, while root apices displaced lingually instead. These small extends of displacement have clinical implications. It is shown that about a 0.2-mm displacement might exert a vertical force about 1 N together with a horizontal force about 1.5 N [13, 20]. By increasing the inclination of teeth, the strain in the periodontal ligament increased from about 37 to 58 mJ. The von Mises stresses around the cervical and apical areas differed for each tooth and each model, without a similar pattern. Increasing the angle of the teeth resulted in much higher cervical stresses in the central incisors, but not in the canines. In the lateral incisor, cervical stress increased until 100° of inclination but reduced to about half by increasing the angle to 110°. Apical stress increased rather consistently in the incisor and lateral incisors, by increasing the inclination. However, in the canines, it reduces to about half, from the first to fourth models. It was previously shown that the act of splinting itself can change the displacement pattern. The reason can be the lack of a telescopic movement in the connection of wire with a composite. Additionally, the pattern of displacement depends on the coordinates of the applied force in relation to the center of resistance of the tooth [8]. This study showed that in patients with upright anterior teeth, the displacement can be lingual, whereas in a patient with an increased IMPA, incisal displacements will be labial while root apices will move towards the lingual direction. Our results warn against long durations of splinting in patients with greater labial inclination of mandibular teeth and/or patients with reduced labial bone thickness, because in such patients the loads might be more of pathologic nature and cause periodontal damage and pathologic tooth mobility [8, 22, 23].

It is not known if bonded lingual retainers have a negative effect on the periodontal tissues [13, 24]. Gingival damage and recession can be caused by numerous factors, among which mechanical trauma and bacterial periodontal disease are the most important ones [13, 25–28]. Besides increasing plaque, these appliances are also criticized for changing the mode of functional loads exerted on the anterior teeth, and compromising the health of periodontium [13, 29–31]. However; the studies regarding the consequences of splinting on the status of periodontium are limited, and no results exist regarding force distributions [13, 29–31]. Many studies have shown no significant evidence regarding any damage caused to periodontium or soft tissues adjacent to teeth, after using fixed lingual retainers even for long durations [9–14, 24]. This level of safety might not change depending on the wire used in the fixed retainer, even in durations as long as 10 years [10]. Nevertheless, using wire diameters that allow for physiologic tooth movement, especially in patients at higher risk for developing periodontal diseases, is recommended, as an ideal bonded retainer should be passive and semi-rigid to maintain physiologic tooth mobility after splinting [5, 13, 32]. Even plaque accumulation following the application of lingual-fixed retainers is questionable [24]. There were also reports of no significant displacement after using fixed retainers [13]. However, a negative effect of bonded retainers on tooth mobility was observed by Watted et al. [15]. Another study as well showed negative effects of long-term fixed retention on periodontal health, although the changes were mostly mild [14]. Also increased gingival recession, increased plaque retention, and bleeding upon probing have been reported in another study [32]. In that study, gingival recession was more advanced in patients with past histories of orthodontic treatment, which might be attributed to previous orthodontic movements and tooth rotations which might have stretched collagen fibers within periodontal and gingival tissues [13, 32–41].

This study was limited by some factors. In vitro studies cannot reproduce the highly dynamic nature of oral environment with occlusal loads rapidly changing in extent and direction. However, there is no alternative to this method, as in vivo studies need to either be on radiographic images (which cannot show the extent of bone loss accurately) or be in animals, which again are irrelevant to human; and none of other options can show the distribution of forces [13, 14, 17]. Moreover, utilization of radiographic and computerized tomography techniques only for the sake of research and without any treatment needs would expose patients to unnecessary doses of carcinogen X-ray and hence are not easily justifiable ethically [42].

## Conclusions

Increasing the labial inclination can mostly harm the central incisors, followed by the lateral incisors. This finding warns against long durations of splinting in patients with increased inclination of the mandibular incisors (i.e., increased IMPA) and/or patients with reduced labial bone thickness.
